# Supplementary material for: From sequence to enzyme mechanism using multi-label machine learning
Source: BMC Bioinformatics. 2014 May 19;15:150. doi: 10.1186/1471-2105-15-150 (PMC4229970; doi:10.1186/1471-2105-15-150)
Supplement: Additional file 2 — Java code of ml2db. Additional file ml2db_code.tar.gz contains the Java source code to run the multi-label machine learning experiments and save the results to database. The code’s Javadoc is included. [file 1471-2105-15-150-S2.zip › additional file 2/ml2db/utils/doc/index-files/index-21.html]

U-Index


JavaScript is disabled on your browser.


- Overview
- Package
- Class
- Use
- Tree
- Deprecated
- Index
- Help

- Prev Letter
- Next Letter

- Frames
- No Frames

- All Classes

A B C D E F G H I J K L M N O P Q R S T U V W X Y 


## U

uk.ac.standrews.utils.main - package uk.ac.standrews.utils.main


uk.ac.standrews.utils.main.collectionutils - package uk.ac.standrews.utils.main.collectionutils


uk.ac.standrews.utils.main.collectionutils.setutils - package uk.ac.standrews.utils.main.collectionutils.setutils


uk.ac.standrews.utils.main.database - package uk.ac.standrews.utils.main.database


uk.ac.standrews.utils.main.diff - package uk.ac.standrews.utils.main.diff


uk.ac.standrews.utils.main.guiutils - package uk.ac.standrews.utils.main.guiutils


uk.ac.standrews.utils.main.maputils - package uk.ac.standrews.utils.main.maputils


uk.ac.standrews.utils.main.setutils - package uk.ac.standrews.utils.main.setutils


uk.ac.standrews.utils.main.stats - package uk.ac.standrews.utils.main.stats


uk.ac.standrews.utils.main.stringutils - package uk.ac.standrews.utils.main.stringutils


uk.ac.standrews.utils.main.webutils - package uk.ac.standrews.utils.main.webutils


uk.ac.standrews.utils.main.webutils.simpledomparser - package uk.ac.standrews.utils.main.webutils.simpledomparser


uk.ac.standrews.utils.test - package uk.ac.standrews.utils.test


uk.ac.standrews.utils.test.database - package uk.ac.standrews.utils.test.database


uk.ac.standrews.utils.test.maputils - package uk.ac.standrews.utils.test.maputils


uk.ac.standrews.utils.test.setutils - package uk.ac.standrews.utils.test.setutils


uk.ac.standrews.utils.test.stats - package uk.ac.standrews.utils.test.stats


uniform(double, double) - Method in class edu.cornell.lassp.houle.RngPack.RandomElement


uniformPick(Vector) - Static method in class uk.ac.standrews.utils.main.stats.UniformRandomUtils


UniformRandomSingleton - Class in uk.ac.standrews.utils.main.stats
:   Class

UniformRandomSingletonTest - Class in uk.ac.standrews.utils.test.stats
:   Class

UniformRandomSingletonTest() - Constructor for class uk.ac.standrews.utils.test.stats.UniformRandomSingletonTest


UniformRandomUtils - Class in uk.ac.standrews.utils.main.stats
:   Utilities for extracting with uniform random distribution

UniformRandomUtils() - Constructor for class uk.ac.standrews.utils.main.stats.UniformRandomUtils


UniformRandomUtilsTest - Class in uk.ac.standrews.utils.test.stats
:   Class

UniformRandomUtilsTest() - Constructor for class uk.ac.standrews.utils.test.stats.UniformRandomUtilsTest


USER\_PROP - Static variable in class uk.ac.standrews.utils.main.database.DbConn


Utils - Class in uk.ac.standrews.utils.main
:   Various utilities to manipulate vectors of numbers, get a timestamp etc.

Utils() - Constructor for class uk.ac.standrews.utils.main.Utils


UTILS\_DATA - Static variable in class uk.ac.standrews.utils.main.PathUtils


UtilsTest - Class in uk.ac.standrews.utils.test
:   Class

UtilsTest() - Constructor for class uk.ac.standrews.utils.test.UtilsTest

A B C D E F G H I J K L M N O P Q R S T U V W X Y

- Overview
- Package
- Class
- Use
- Tree
- Deprecated
- Index
- Help

- Prev Letter
- Next Letter

- Frames
- No Frames

- All Classes
